# Cardiac Protection after Systemic Transplant of Dystrophin Expressing Chimeric (DEC) Cells to the *mdx* Mouse Model of Duchenne Muscular Dystrophy

**DOI:** 10.1007/s12015-019-09916-0

**Published:** 2019-10-15

**Authors:** Maria Siemionow, M. Malik, P. Langa, J. Cwykiel, S. Brodowska, A. Heydemann

**Affiliations:** 1 Department of Surgery, Poznan University of Medical Sciences, Poznan, Poland; 2 Department of Orthopaedics, University of Illinois at Chicago, Chicago, IL 60612 USA; 3 Department of Physiology and Biophysics, University of Illinois at Chicago, Chicago, IL USA; 4 Center for Cardiovascular Research, University of Illinois at Chicago, Chicago, IL USA

**Keywords:** (<10): Duchenne muscular dystrophy, Stem cells, Myoblasts, MSC, Dystrophin expressing chimeric (DEC) cells, DEC therapy, *mdx* mice, Systemic DEC transplant, Cardiac protection, Echocardiography

## Abstract

Duchenne Muscular Dystrophy (DMD) is a progressive lethal disease caused by X-linked mutations of the dystrophin gene. Dystrophin deficiency clinically manifests as skeletal and cardiac muscle weakness, leading to muscle wasting and premature death due to cardiac and respiratory failure. Currently, no cure exists. Since heart disease is becoming a leading cause of death in DMD patients, there is an urgent need to develop new more effective therapeutic strategies for protection and improvement of cardiac function. We previously reported functional improvements correlating with dystrophin restoration following transplantation of Dystrophin Expressing Chimeric Cells (DEC) of myoblast origin in the *mdx* and *mdx/scid* mouse models. Here, we confirm positive effect of DEC of myoblast (MB^*wt*^/MB^*mdx*^) and mesenchymal stem cells (MB^*wt*^/MSC^*mdx*^) origin on protection of cardiac function after systemic DEC transplant. Therapeutic effect of DEC transplant (0.5 × 10^6^) was assessed by echocardiography at 30 and 90 days after systemic-intraosseous injection to the *mdx* mice. At 90 days post-transplant, dystrophin expression in cardiac muscles of DEC injected mice significantly increased (15.73% ± 5.70 –MB^*wt*^/MB^*mdx*^ and 5.22% ± 1.10 – MB^*wt*^/MSC^*mdx*^ DEC) when compared to vehicle injected controls (2.01% ± 1.36) and, correlated with improved ejection fraction and fractional shortening on echocardiography. DEC lines of MB and MSC origin introduce a new promising approach based on the combined effects of normal myoblasts with dystrophin delivery capacities and MSC with immunomodulatory properties. Our study confirms feasibility and efficacy of DEC therapy on cardiac function and represents a novel therapeutic strategy for cardiac protection and muscle regeneration in DMD.

## Introduction

Duchenne Muscular Dystrophy (DMD) is an X-linked neuromuscular disorder caused by a mutation in the dystrophin gene. Dystrophin is a vital structural link between the extracellular matrix and the cytoskeletal proteins and plays an essential role in several important biochemical extracellular signaling pathways. Dystrophin deficiency clinically manifests as skeletal and cardiac muscle weakness as myofibrils undergo damage, inflammation, and fibrosis [1]. Cardiomyopathy is a significant cause of morbidity and mortality in DMD patients [2–4]. Histopathological evidence suggests that the fibro-fatty replacement of cardiomyocytes is a significant pathophysiological mechanism in the development of cardiomyopathy in the *mdx* mice [5–7]. Specifically, studies on echocardiographic assessment confirm increases in the left ventricular posterior wall thickness in the *mdx* mice when compared to the wild type controls, presumably due to fibrous deposition. Current treatments of DMD related cardiomyopathy have aimed to decrease cardiac mortality by preventing cardiac fibrosis [4, 8–10]. Several studies have shown that common blood pressure medications such as angiotensin-converting enzyme inhibitors, aldosterone antagonists, and angiotensin receptor blockers decrease myocardial fibrosis and improve circumferential strain in DMD mouse models [11–14]. Chronic steroid treatment correlated with a smaller age-related increase in the myocardial fibrosis burden in the *mdx* mouse model [13]. Despite the evidence suggesting that these treatments confer an anti-fibrotic effect on the cardiomyocytes in the ventricular walls of *mdx* mice, novel therapies are needed to exert a protective effect and restore cardiac function to clinically appreciable levels [11–15].

Cellular level gene therapies are emerging as innovative approaches for researchers to target DMD related cardiac manifestations, which are often the most devastating aspects of disease progression. These new cellular therapies offer the potential to cure DMD, including the cardiomyopathy characteristics [16–23]. Putten et al. demonstrated that cardiac myocyte dystrophin levels as low as 4–15% of wild type mice can delay or even partially ameliorate the effects of cardiomyopathy in the *mdx* mice [24]. Several potential gene therapies aiming at dystrophin restoration such as exon skipping, gene editing via viral vectors, and gene slicing CRISPR system delivered by adeno-associated viruses have been described in the literature, but their efficacy in dystrophin restoration for the cardiomyocytes to clinically relevant levels has been limited [16–23, 25].

Stem cell transplants based on the delivery of either autologous or allogenic stem cells have shown promise as an alternative method for DMD treatment, but limited or short-term cell engraftment, allogenic immune response, and side effects of immunosuppressive therapy have all been challenges that these treatments have faced in their path to changing the course of the disease for DMD patients [26–39].

Previous in vitro studies have shown that autologous multipotent stem cells from wild type mice are able to differentiate into reconstituted skeletal muscle cells when injected into *mdx* mice [32]. Gussoni et al. demonstrated that bone marrow transplantation via intravenous injection of hematopoietic stem cells can reconstitute expression of dystrophin in affected animals [40]. Allogeneic stem cell transplantation of satellite cells, mesenchymal stem cells, adipose mesenchymal stem cells bone marrow, pericytes, and iPS demonstrated dystrophin expression in small and large animal models of DMD with variable results [26–28, 31–37, 39–42].

The success of stem cell engraftment is limited by the allogenic immune response [27, 35, 37, 38, 43–46]. Thus, immunosuppressive therapy was used to support the engraftment, however the efficacy remained sub-optimal [27, 34, 35, 37, 39, 47]. It is clear that new approaches are needed in order to enhance and maintain cell engraftment in DMD stem cell-based therapies [29, 48, 49].

We have previously reported in both the *mdx* and *mdx/scid* mouse models of DMD that local transplantation of Dystrophin Expressing Chimeric (DEC) cells significantly improved dystrophin expression which correlated with significant improvement of strength and functional outcomes [31, 32]. Thus, in order to assess the effect of DEC therapy on cardiac function, we applied our established protocol of systemic-intraosseous administration of both DEC lines (MB^*wt*^/MB^*mdx*^ and MB^*wt*^/MSC^*mdx*^) into *mdx* mice [31, 32, 45].

In this study, for the first time we have examined the effect of systemic-intraosseous administration of DEC cell therapy of myoblast (MB) and mesenchymal stem cell (MSC) origin and assessed changes in the cardiophysiologic parameters by echocardiography.

Since the *mdx* mouse model of DMD manifests a less aggressive form of the disease before age of 10 months [9, 10] this allows the study pathophysiological mechanisms in response to different therapies. Echocardiography in mice has proven to be an accurate and thorough method to assess global left ventricular function in the *mdx* mice. In order to assess the effect of fused (DEC) and non-fused myoblasts and MSC on the cardiac function in 6–8 week old mdx mice, we applied echocardiography at day 0 and at 30 days, and 90 days after systemic administration of DEC to the bone marrow compartment of the *mdx* mice. This allowed for assessment of DEC therapy effects on mdx mice at 5 months of age, prior to the onset of global cardiac dysfunction, thus it was possible to check if there is a protective therapeutic effect after systemic DEC delivery.

## Materials and Methods

### Experimental Animals

This study was approved by the Institutional Animal Care and Use Committee (IACUC) of the University of Illinois at Chicago, which is accredited by the American Association for the Accreditation of Laboratory Animal Care (AAALAC). All animals received humane care in compliance with the ‘Principles of Laboratory Animal Care’ formulated by the National Society for Medical Research and the ‘Guide for the Care and Use of Laboratory Animal Resources’ published by the US National Institutes of Health. Six to eight-week old male *mdx* mice (C57BL/10ScSn-Dmd^mdx^/J, stock number 001801) and the corresponding background wild type (wt) mice (C57BL/10ScSnJ, stock number 000476) were purchased from Jackson Laboratories. Mice were housed in the Molecular Biology Research Building, an AAALAC-accredited animal facility, at University of Illinois at Chicago.

### Creation of Murine Dystrophin Expressing Chimeric (DEC) Cells of Myoblast and Mesenchymal Stem Cells Origin

#### Myoblast Isolation

DEC cells of myoblast origin were created after myoblast isolation from the *mdx* and *snj* wild type (wt) animals as reported before [32]. Briefly, hind limb muscles were harvested, minced and incubated with 1.5 U/l Collagenase type D (Roche–ThermoFischer, Waltham, MA, USA) and 2.4 U/l Dispase II (Sigma, St. Louis, MO, USA) in 2.5 mM CaCl_2_ solution at 37 °C for 30 min. Using Primary Culture Media (F-10+), Ham’s F-10 medium (Gibco-ThermoFischer, Waltham, MA, USA) supplemented with 20% Fetal Bovine Serum (Gemini Bio-Products, West Sacrament, CA, USA), 1% Antimycotic/antibacterial solution (Gibco- ThermoFischer, Waltham, MA, USA) and 50 μl of 25 μg/ml basic fibroblast growth factor (bFGF, Peprotech, Rocky Hill, NJ, USA), samples were suspended and digested tissues were further mechanically dissociated. Tissue homogenates were filtered through 100 μm and subsequently 70 μm pore size nylon meshes (ThermoFischer, Waltham, MA, USA). The samples were centrifuged (350 g, 5 min) and washed twice with F-10-based primary culture media. After centrifugation, cells were counted and plated in 75 cm^2^ collagen-coated tissue flasks (Celltreat Scientific Product, Pepperell, MA, USA). Upon 60–80% of confluence, adherent cells were harvested with mechanical agitation in DPBS and pre-plated for 15 min in collagen-coated flask to eliminate fibroblasts from culture. Non-adherent cells were transferred for further culture in F-10-based Primary Culture Media. After 3–4 additional passages and pre-plating steps, myoblasts were expanded in F10/DMEM-based Myoblast Growth Medium supplemented with 20% Fetal Bovine Serum (Gemini Bio-products, West Sacramento, CA, USA), 1% Antimycotic/antibacterial solution (Gibco- ThermoFischer, Waltham, MA, USA) and 50 μl of 25 μg/ml basic fibroblast growth factor (bFGF, Peprotech). During expansion, myoblast (MB) harvests were performed with 0.25% Trypsin-EDTA solution (Gibco- ThermoFischer, Waltham, MA, USA) as described above. Murine MB, passages 4–6, were used for fusion procedure.

#### Mesenchymal Stem Cells Isolation

MSC were harvested from bone marrow cavities as described previously [43]**.** Briefly, MSC were harvested from the femur and tibia bones of 6 weeks old *mdx* mice by flushing technique. Next, cells were washed and cultured in media composed of 40% alpha Modified Eagle Medium (αMEM), 40% F-12 nutrient mixture (Invitrogen), 10% fetal bovine serum (FBS, Gemini Bio Products, Atlanta, USA), and 1X antibiotic-antimycotic solution (Invitrogen). The non-adherent cell population was removed after 72 h and the adherent cells were washed with fresh media and cultured for 7 days. Harvested cells were depleted (Miltenyi) with antibodies to CD11b and CD45 (eBiosciences). CD45-CD11b- subpopulation of adherent cells were cultured in 175 cm^2^ flasks (Nunc) at a density of 1 × 10^6^ cells per flask. After 14 days of culturing, MSC phenotype was confirmed by flow cytometry (CD105, CD29, CD44, Sca-1). Murine MSC, passages 4–6 were used for fusion procedure.

#### Cell Fusion Procedure

**Fig. 1:**
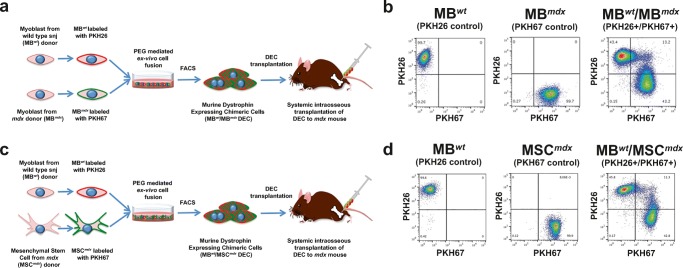
**Diagram presenting polyethylene glycol (PEG) mediated ex-vivo cell fusion procedure to create murine Dystrophin Expressing Chimeric Cell (DEC) and systemic-intraosseous transplantation of murine DEC to the*mdx*mice. a** Ex vivo created DEC derived from the wild type *snj* myoblasts (MB^*wt*^) and dystrophin-deficient *mdx* (MB^*mdx*^) mice. **b** Representative flow cytometry plots presenting fusion of the PKH26 labeled MB^*wt*^ and PKH67 labeled MB^*mdx*^ parent myoblasts assessed by FACS. The overlapping fluorescence of PKH26/PKH67 confirms chimeric state for MB^*wt*^/MB^*mdx*^ DEC cell line (Siemionow M. et al. Stem Cell Rev. 2018 Apr;14(2):189–199 [32], http://creativecommons.org/licenses/by/4.0/). **c** Ex-vivo created DEC derived from the wild type *snj* myoblasts (MB^*wt*^) and mesenchymal stem cells from dystrophin-deficient *mdx* (MSC^*mdx*^) mice. **d** Representative flow cytometry plots presenting fusion of the PKH26 labeled MB^*wt*^ and PKH67 labeled MSC^*mdx*^ parent cells assessed by FACS. The overlapping fluorescence of PKH26/PKH67 confirms chimeric state for MB^*wt*^/MSC^*mdx*^ DEC cell line

Our standard polyethylene glycol (PEG) fusion protocol was applied as described before [31, 32]**.** Parent *snj* (wt*)* and *mdx* myoblasts (MB^*wt*^ and MB^*mdx*^) as well as *mdx* MSC (MSC^*mdx*^) were harvested and washed in serum-free DMEM culture media supplemented with 1% Antibiotic-Antimycotic solution (Gibco-ThermoFischer, Waltham, MA, USA). Prior to fusion, parent myoblasts for MB^*wt*^/MB^*mdx*^ DEC fusion and myoblast and MSC for MB^*wt*^/MSC^*mdx*^ DEC fusion were fluorescently labeled using either PKH26 or PKH67 (Sigma, St. Louis, MO, USA) tracking membrane dyes according to manufacturer’s instruction. After the pellet was mobilized, the fusion procedure was performed using fusion medium consisting of 3.5 g of polyethylene glycol (PEG 4000, EMD), 400 μl of DMSO (Sigma) and 2 ml of serum-free DMEM basal medium supplemented with 1X Antibiotic/Antimycotic solution. The fused cells were then washed in complete culture media and transferred to PBS-based fluorescently activated cells sorting (FACS) buffer containing 5% HEPES, 1% EDTA and 5% FBS. Finally, cells presenting double (PKH26/PKH67) staining were selected by FACS (BD Astrios, BD Biosciences) and used for further in vitro analysis or transplanted to the recipient *mdx* mice. The experimental study design is outlined on Fig. 1a, c. A total of 7 fusions (*n* = 7) were performed to create murine MB^*wt*^/MB^*mdx*^ and MB^*wt*^/MSC^*mdx*^ DEC lines to characterize DEC in vitro and to assess DEC efficacy in vivo after systemic intraosseous transplant to the *mdx* mice.

#### FACS Analysis for Confirmation of DEC Fusion

Following fusion, samples of sorted PKH26/PKH67 labeled DEC, as well as corresponding single stained controls (PKH26 labeled MB^*wt*^, PKH67 labeled MB^*mdx*^ and PKH67 labeled MSC^*mdx*^) and unstained controls (MB^*wt*^*,* MB^*mdx*^ and MSC^*mdx*^) were fixed with 4% paraformaldehyde for 15 min. Samples were re-suspended in 1X DPBS and analyzed via flow cytometry (Gallios, Beckman Coulter, San Jose, CA, USA).

#### Systemic DEC Transplant and Experimental Design

Dystrophin Expressing Chimeric cells were prepared for systemic-intraosseous injections to the femoral bone of the *mdx* mice as previously reported [43, 45, 46]. Briefly, DEC were counted and washed in sterile saline and viability was assessed with 0.4% Trypan Blue. A single DEC dose was suspended in 60 μl of total volume of saline and transferred to tuberculin syringe with 27G needle (ThermoFischer, Waltham, MA, USA). *Mdx* recipients were anesthetized with 1.5% isoflurane inhalation and injected with subcutaneous buprenorphine SR lab (0.1 mg/kg) for analgesia. Following a 5-mm skin incision over the mid-femoral level, the subcutaneous tissue and the overlying muscles were dissected. The opening was made in the femoral bone with 28G needle passing through the cortex into the medullary cavity of the femur and 60 μl of the recipient bone marrow were aspirated prior to DEC injection. Next, DEC cells and controls were suspended in 60 μl of sterile saline solution and were injected into the femoral bone marrow cavity in the respective experimental groups. After injection, the bone was sealed with bone wax to prevent cell leakage. The muscle and skin were closed using 4–0 monofilament absorbable suture. Animals were allowed to recover in a heated environment and promptly returned to the colony.

After age-matching the recipient *mdx* mice were randomized into the following experimental groups: vehicle control (*n* = 3**,** 60 μl saline), not-fused MB^*wt*^ and MB^*mdx*^ (n = 3, 0.25 × 10^6^/donor–total 0.5 × 10^6^ in 60 μl saline), fused MB^*wt*^/MB^*mdx*^DEC (*n* = 4, 0.5 × 10^6^ in 60 μl saline), not-fused MB^*wt*^ and MSC^*mdx*^ (n = 3 0.25 × 10^6^ /donor–total 0.5 × 10^6^ in 60 μl saline), and fused MB^*wt*^/MSC^*mdx*^ DEC (n = 3, 0.5 × 10^6^ in 60 μl saline). Animal follow-up consisted of in vivo assessments of echocardiography at day 0 before DEC injection and at 30 and 90 days post-DEC transplant. At day 90 endpoint the animals were euthanized and heart muscles were harvested for histological and immunofluorescence analysis.

#### Histological and Immunofluorescence Analysis of Cardiac Muscle Cross-Sections

For histological analysis of heart muscles paraffin blocks were cut at 5-μm sections. Samples were deparaffinized and subsequently stained with hematoxylin-eosin (Abcam, ab245880, Cambridge, MA, USA) and mounted (Poly-Mount, PolySciences Inc., Warrington, PA, USA) to analyze heart muscle structure. A BX51/IX70 Microscope (Olympus, Japan) was used for imaging.

For immunofluorescence analysis, OCT embedded frozen heart samples were cut with a cryotome (ThermoFischer, Waltham, MA, USA) at 5-μm cross-sections. Samples were fixed with ice-cold acetone for 8 min. Blocking was performed with 10% normal goat serum in 1% BSA for 60 min. Dystrophin was detected using primary rabbit anti-dystrophin antibody (1:50, ab15277, Abcam, Cambridge, MA, USA) and secondary goat anti-rabbit Alexa Fluor 488-conjugated secondary antibody (1:500, A11001, Life Technologies, USA). Nuclei were counterstained with DAPI (ab104139, Abcam, Cambridge, MA, USA). A Zeiss Meta confocal microscope with ZEN software (Carl Zeiss, Oberkochen, Germany) was used for fluorescence signal detection and ImageJ for analysis. The number of dystrophin-positive muscle fibers in five standardized regions of each cross-section were counted and normalized to total number of fibers; two non-serial cross-sections were quantified for each animal group (*n* > 2, mean ± SD, one-way ANOVA with post-hoc Tukey test) at day 90.

#### Echocardiography Assessment

Echocardiography was performed with a VEVO 2100 system (VisualSonics, Inc., Toronto, Ontario, Canada) and a 30-MHz cardiac probe (RMV707B). The *mdx* mice systemically injected with either saline, not-fused MB^*wt*^ and MB^*mdx*^, not-fused MB^*wt*^ and MSC^*mdx*^ or fused (MB^*wt*^/MB^*mdx*^ or MB^*wt*^/MSC^*mdx*^) DEC therapies were followed and scanned at day 0 before DEC injection and at day 30 and 90 after DEC transplant. For each echocardiographic recording, we optimized the sweep speed, depth, focus, and gain settings. Mice were individually placed in an induction chamber receiving 1.5% isoflurane in 100% oxygen at a constant flow rate of 1 L/min. Once anesthetized, mice were removed from the induction chamber and placed in the supine position on the preheated ultrasound stage. Electrode gel was applied to the paws, which were taped down onto the electrocardiograph (ECG) electrodes of the scanner to monitor the heart rate, which was between 350 and 450, throughout the test. Anesthesia was maintained with 1%–1.5% isoflurane in 100% oxygen at a flow rate of 1 L/min, and body temperature was maintained at 37 °C to ensure that heart function was not depressed as a result of the anesthetic regime [50].

Two-dimensional M-mode echocardiographic images were obtained from the long-axis brightness B-mode view through the center of the left ventricle. The M-mode line was drawn close to the base of the heart towards the apex. The left ventricular dimensions (end-diastolic diameter and end-systolic diameter, denoted as LVEDD and LVESD, respectively), left ventricular volume (end-diastolic volume and end-systolic volume, denoted as LVEDV and LVESV, respectively) and heart rate (HR) were measured from 4.95 s cine images acquired from M-Mode tracings. From these measurements, stroke volume, ejection fraction, fractional shortening and cardiac output were calculated using instructions outlined from the American Society of Echocardiography [9, 10, 13, 43, 51, 52].

Ejection fraction (EF) is defined as: EF = [(LVEDV – LVESV) / LVEDV] × 100. Cardiac output (CO) is defined as: CO = SV x HR, where SV is stroke volume and HR is heart rate. Stroke volume (SV), defined as the volume of blood pumped by the heart in one cycle, was calculated as: SV = LVEDV – LVESV.

Fractional shortening (FS) is defined by the following formula: FS = [(LVEDD – LVESD) / LVEDD] × 100, where LVEDD is left ventricle end-diastolic diameter and LVESD is left ventricle end-systolic diameter.

### Statistical Analysis

Data were expressed as mean ± SD. GraphPrism and Microsoft Excel software were used to perform statistical analysis of echocardiography data. One-way ANOVA for dystrophin-positive fibers, and day 0, day 30, and day 90 echocardiography measurements with Tukey post-hoc test for pairwise comparisons were used to define statistical significance. *P* values were considered significant below 0.05.

## Results

### Confirmation of Myoblast and MSC Fusion and Creation of Murine Dystrophin Expressing Chimeric (DEC) Cell Lines

We applied our well-established ex-vivo PEG mediated cell fusion procedure and confirmed fusion efficacy by flow cytometry (Fig. 1) [31, 32]. To confirm fusion, suitable controls of unstained myoblasts derived from wild type mice (MB^*wt*^) and MB (MB^*mdx*^) and MSC (MSC^*mdx*^) derived from the dystrophin deficient *mdx* mice as well as single stained controls of PKH26 labeled MB^*wt*^ and PKH67 labeled MB^*mdx*^ for confirmation of fusion for DEC of myoblast origin (Fig. 1b) and PKH26 labeled MB^*wt*^ and PKH67 labeled MSC^*mdx*^ for confirmation of fusion for DEC of MSC origin were used to determine localization of the double stained PKH26/PKH67 cells (Fig. 1d).

### DEC Therapy Increases Dystrophin Expression and Decrease in Cardiac Muscle Fibrosis at 90 Days after Systemic DEC Transplant to the *mdx* Recipient Mouse

**Fig. 2:**
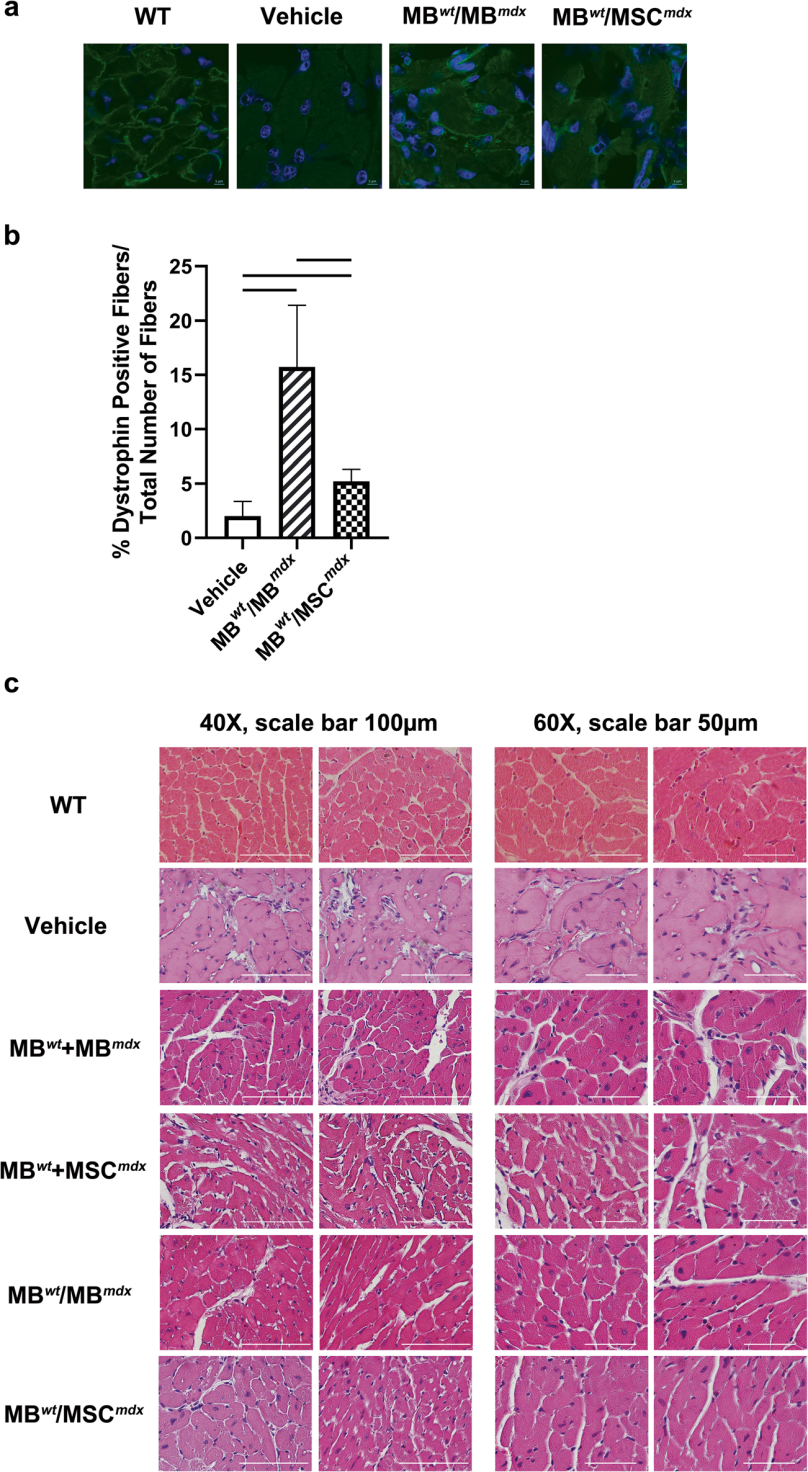
**Significant increase of dystrophin expression and decrease in cardiac muscle fibrosis at 90 days after systemic-intraosseous DEC transplant to the*mdx*mice. a** Representative immunofluorescence images presenting restoration of the dystrophin (green) in the cardiac muscles of the DEC (MB^*wt*^/MB^*mdx*^ and MB^*wt*^/MSC^*mdx*^) injected *mdx* hosts compared to the vehicle injected *mdx* controls and wild type control (*n* > 2). Nuclei were counterstained with DAPI (blue); Magnification 120X, scale bar 5 μm (ZEISS 710 META, Oberkochen, Germany). **b** Quantification of dystrophin-positive muscle fibers for both DEC lines: MB^*wt*^/MB^*mdx*^ (15.73% ± 5.70, *p* < 0.0001, *n* = 4) and MB^*wt*^/MSC^*mdx*^ (5.22% ± 1.10, *p* = 0.0406, *n* = 2) in cardiac muscle at 90 days after intraosseous-systemic DEC transplant when compared to the vehicle injected controls (2.01% ± 1.36, n = 2)(mean ± SD); significance between MB^*wt*^/MB^*mdx*^ and MB^*wt*^/MSC^*mdx*^ (p < 0.0001). Dystrophin-positive fibers count was normalized to the total number of fibers counted within the region of interest (mean ± SD, *p* < 0.05, one-way ANOVA with post-hoc Tukey test). **c** Representative images of hematoxylin-eosin (H&E) stained cross-sections of cardiac muscle of *mdx* mice confirming decrease in cardiac muscle fibrosis in MB^*wt*^/MB^*mdx*^ (n = 2) and MB^*wt*^/MSC^*mdx*^ (n = 2) DEC injected *mdx* hosts compared to the vehicle injected, not-fused MB^*wt*^ + MB^*mdx*^ (*n* = 1) and not-fused MB^*wt*^ + MSC^*mdx*^ (n = 2) injected controls; Magnification 40X, scale bar 100 μm (left panel); magnification 60X, scale bar 50 μm (right panel)

The efficacy of DEC engraftment was confirmed by restoration of dystrophin expression at 90 days after systemic-intraosseous transplantation of both DEC lines (0.5 × 10^6^) of myoblast and MSC origin (MB^*wt*^/MB^*mdx*^ and MB^*wt*^/MSC^*mdx*^) to the *mdx* recipient mice. Immunofluorescence staining confirmed long-term engraftment and dystrophin expression (dystrophin-positive fibers) in MB^*wt*^/MB^*mdx*^ and MB^*wt*^/MSC^*mdx*^ DEC injected animals when compared to the vehicle injected *mdx* control mice (Fig. 2a, b). Quantification of dystrophin-positive fibers at 90 days after DEC transplant confirmed significant increase in dystrophin expression in heart samples of MB^*wt*^/MB^*mdx*^ (15.73% ± 5.70, *p* < 0.0001) and the MB^*wt*^/MSC^*mdx*^ (5.22% ± 1.10, *p* = 0.0406) DEC injected *mdx* mice as compared to the vehicle injected *mdx* control (2.01% ± 1.36) and between the groups MB^*wt*^/MB^*mdx*^ and the MB^*wt*^/MSC^*mdx*^ (p < 0.0001) (Fig. 2b).

Structural analysis of hematoxylin & eosin (H&E) stained cross-section of heart samples revealed decrease in fibrosis in cardiac muscle of mice injected with MB^*wt*^/MB^*mdx*^ or MB^*wt*^/MSC^*mdx*^ DEC lines when compared to the *mdx* mice injected with vehicle and not-fused MB^*wt*^ + MB^*mdx*^ and MB^*wt*^ *+* MSC^*mdx*^ (Fig. 2c).

### Functional Outcomes Assessed by Echocardiography Confirm Cardiac Protection at 90 Days after Systemic DEC Transplant to the *mdx* Mice

**Fig. 3:**
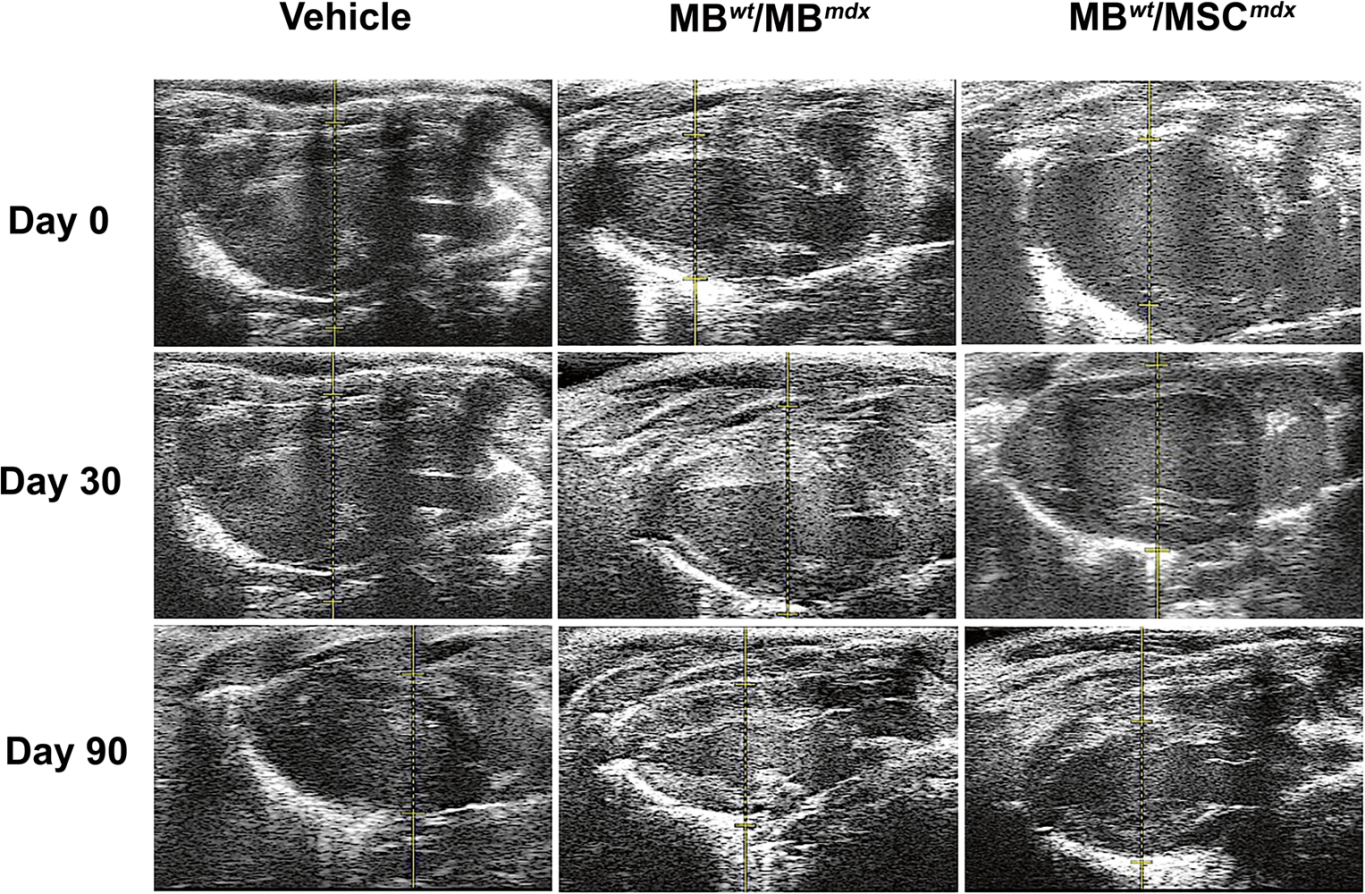
**Confirmation of reduced cardiac disease in*mdx*mice at 90 days after systemic-intraosseous DEC (MB**^***wt***^**/MB**^***mdx***^**DEC and MB**^***wt***^**/MSC**^***mdx***^**) transplant confirmed by B-mode echocardiography.** B-Mode image of parasternal long axis view of left ventricle of *mdx* vehicle control (left column) and *mdx* mice systemically injected with MB^*wt*^/MB^*mdx*^ DEC (middle column) and MB^*wt*^/MSC^*mdx*^ (right column) (Vevo 2100, VisualSonics). Day 0, 30, and 90 B-mode echo images of *mdx* vehicle controls demonstrate a trend of posterior wall disorganization and thickening. Though a degree of disorganization and thickening still occurs in both MB^*wt*^/MB^*mdx*^ and MB^*wt*^/MSC^*mdx*^ DEC treated mice, this effect appears dampened compared to the vehicle controls across the same corresponding time points

**Fig. 4:**
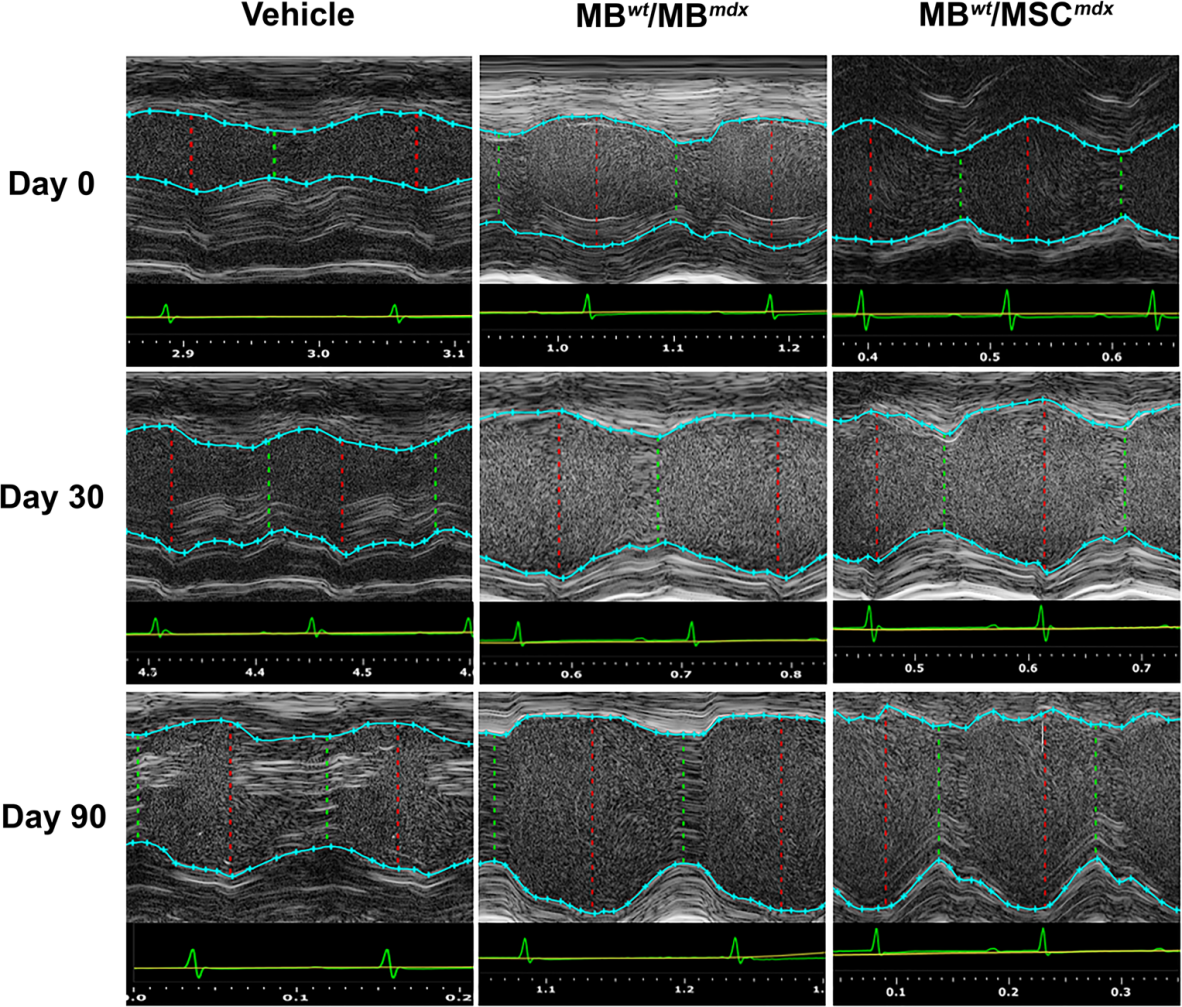
**Confirmation of reduced cardiac disease in*mdx*mice at 90 days after systemic-intraosseous DEC (MB**^***wt***^**/MB**^***mdx***^**DEC or MB**^***wt***^**/MSC**^***mdx***^**) transplant confirmed by M-mode echocardiography.** Echocardiographic M-mode image of parasternal long axis view of anterior and posterior walls of the left ventricle of the *mdx* vehicle control (left column) and *mdx* mice systemically treated with MB^*wt*^/MB^*mdx*^ DEC (middle column) and with MB^*wt*^/MSC^*mdx*^ DEC (right column) (Vevo 2100, VisualSonics). LV tracings are shown as the light blue line connecting light blue crosses. Tracings of both the anterior and posterior left ventricular walls were performed on the first continuous white line that could be followed across the entirety of a representative M-mode image. The red dashed lines represent the left ventricle at the end of systole, while the dashed green lines represent the ventricle during diastole. The white, wavy lines below the inferior tracings represent the myocardial fibers of the posterior ventricular wall. At 90 days post-transplant, vehicle injected mice demonstrated increase in disarray of fiber orientation as well as gross thickening of the posterior left ventricular wall and diminished left ventricular chamber size compared to both DEC treated groups

Long-axis brightness B-mode (Fig. 3) view through the center of the left ventricle was used to perform echocardiography M-mode (Fig. 4) for the assessment of nine parameters using the VEVO Lab software. These parameters are summarized in Tables 1 and 2 including: LVESD (left ventricular end systolic diameter), LVEDD (left ventricular end diastolic diameter), LVESV (left ventricular end systolic volume), LVEDV (left ventricular end diastolic volume), SV (stroke volume), HR (heart rate), EF (ejection fraction), FS (fractional shortening) and CO (cardiac output). One-way ANOVA analysis at 90 days post-DEC transplant confirmed significant differences between the fused cell treatment group and the vehicle controls. In addition, ejection fractions (*p* = 0.008) and fractional-shortening (*p* = 0.0129) at day 30 post-transplant were significantly different between the DEC cell lines and the vehicle injected control groups (Table 2).

**Table 1:** Echocardiographic assessment of morphometric cardiac parameters at day 0 and in response to DEC therapy at 30 and 90 days after systemic DEC transplant in *mdx* mice

Experimental group/ Timepoint	Morphometric Parameters			
	**LVESD (mm)**	**LVEDD (mm)**	**LVESV (μl)**	**LVEDV (**μl**)**
Day 0				
Vehicle (*n* = 3)	1.75 ± 0.32	3.18 ± 0.36	9.46 ± 4.61	40.89 ± 11.65
MB^wt^ + MB^mdx^ (n = 3)	1.76 ± 0.05	2.96 ± 0.03	10.83 ± 0.79	31.50 ± 1.41
MB^wt^ + MSC^mdx^ (n = 3)	1.71 ± 0.17	2.65 ± 0.40	10.92 ± 0.99	31.51 ± 2.94
MB^wt^/MB^mdx^ (*n* = 4)	2.02 ± 0.57	3.25 ± 0.61	14.52 ± 10.5	44.35 ± 19.28
MB^wt^/MSC^mdx^ (n = 3)	0.78 ± 0.73	1.88 ± 1.06	2.51 ± 3.84	15.27 ± 18.09
Day 30				
Vehicle (n = 3)	1.01 ± 0.59	2.33 ± 0.14	3.68 ± 3.48	18.81 ± 2.86
MB^wt^ + MB^mdx^ (n = 3)	1.71 ± 0.13	2.55 ± 0.15	9.31 ± 1.03	24.51 ± 0.22
MB^wt^ + MSC^mdx^ (n = 3)	1.86 ± 0.14***†***	2.77 ± 0.16***†***	10.12 ± 1.12	26.64 ± 0.24
MB^wt^/MB^mdx^(n = 4)	2.40 ± 0.49***‡***	3.28 ± 0.31***†‡***	21.28 ± 11.43***‡***	44.01 ± 10.41***‡***
MB^wt^/MSC^mdx^ (n = 3)	1.48 ± 0.82***‡***	2.00 ± 0.93	9.14 ± 8.30	23.90 ± 19.21***‡***
Day 90				
Vehicle (n = 3)	2.49 ± 0.52	3.29 ± 0.60	23.82 ± 11.48	46.10 ± 19.42
MB^wt^ + MB^mdx^ (n = 3)	1.63 ± 0.30	2.60 ± 0.31	9.09 ± 4.49	28.75 ± 8.72
MB^wt^ + MSC^mdx^ (n = 3)	1.79 ± 0.33	2.86 ± 0.34	9.99 ± 4.93	31.59 ± 9.58
MB^wt^/MB^mdx^ (n = 4)	2.03 ± 0.26	2.91 ± 0.29	13.62 ± 4.42	32.85 ± 8.20
MB^wt^/MSC^mdx^ (n = 3)	1.76 ± 0.05	2.89 ± 0.32	9.16 ± 0.66	32.61 ± 8.62

LVESD = left ventricular end systolic diameter, LVEDD = left ventricular end diastolic diameter, LVESV = left ventricular end systolic volume; LVEDV = left ventricular end diastolic volume; All values expressed as mean ± standard error; **†** Significant increase (p < 0.05) compared to vehicle group; **‡** Significant increase (*p* < 0.05) compared to corresponding non-fused cell group; **†‡** Significant increase (p < 0.05) compared to vehicle and corresponding non-fused cell group

**Table 2:** Echocardiographic assessment of functional cardiac parameters at day 0 and in response to DEC therapy at 30 and 90 days after systemic DEC transplant in *mdx* mice

Experimental group/ Timepoint	Functional Parameters				
	**HR (bpm)**	**SV (μl)**	**EF (%)**	**FS (%)**	**CO ml/min**
Day 0					
Vehicle (*n* = 3)	423.98 ± 76.08	31.43 ± 7.08	77.65 ± 4.29	45.17 ± 3.69	12.97 ± 0.43
MB^wt^ + MSC^mdx^ (n = 3)	416.95 ± 38.53	23.32 ± 1.16	73.32 ± 7.24	44.36 ± 4.10	8.80 ± 0.34
MB^wt^/MB^mdx^ (n = 4)	408.79 ± 89.98	29.83 ± 10.39	69.46 ± 9.99	38.45 ± 7.55	11.56 ± 2.36
MB^wt^/MSC^mdx^ (n = 3)	396.24 ± 54.83	12.76 ± 14.28	76.27 ± 10.46	58.44 ± 14.57	5.66 ± 6.26
Day 30					
Vehicle (n = 3)	402.97 ± 58.45	15.13 ± 6.34	67.08 ± 12.00	54.90 ± 27.99	6.46 ± 3.44
MB^wt^ + MB^mdx^ (n = 3)	437.64 ± 26.37	15.99 ± 0.76	60.26 ± 2.63	34.26 ± 1.85	7.36 ± 0.35
MB^wt^ + MSC^mdx^ (n = 3)	475.70 ± 28.7	17.38 ± 0.83	65.32 ± 3.15	33.87 ± 1.78	8.00 ± 0.39
MB^wt^/MB^mdx^ (n = 4)	489.75 ± 20.50	22.73 ± 4.60	53.51 ± 14.53	27.35 ± 8.95	11.88 ± 2.59
MB^wt^/MSC^mdx^ (n = 3)	372.54 ± 12.40	8.00 ± 6.18	61.40 ± 15.01	31.48 ± 9.35	6.62 ± 3.17
Day 90					
Vehicle (n = 3)	493.50 ± 36.00	22.28 ± 7.94	49.98 ± 3.82	24.55 ± 1.91	8.20 ± 0.61
MB^wt^ + MB^mdx^ (n = 3)	446.19 ± 16.06	19.66 ± 4.26	50.46 ± 2.08	29.35 ± 1.52	9.69 ± 2.47
MB^wt^ + MSC^mdx^ (n = 3)	490.32 ± 17.65	21.61 ± 4.70	55.45 ± 2.29	33.99 ± 3.73	10.65 ± 2.72
MB^wt^/MB^mdx^ (n = 4)	511.16 ± 35.85	19.22 ± 3.90	59.07 ± 3.59***†***	30.16 ± 2.22***†***	9.80 ± 1.92
MB^wt^/MSC^mdx^ (n = 3)	469.20 ± 10.97	23.45 ± 7.96	70.39 ± 5.80***†‡***	37.14 ± 3.56***†***	10.91 ± 3.48

SV = stroke volume, HR = heart rate, EF = ejection fraction, FS = fractional shortening, CO = cardiac output; All values expressed as mean ± standard deviation; **†** Significant increase (p < 0.05) compared to vehicle group; **‡** Significant increase (p < 0.05) compared to corresponding non-fused cell group; **†‡** Significant increase (p < 0.05) compared to vehicle and corresponding non-fused cell group

### Changes in Morphometric Parameters at 30 Days after Systemic DEC Transplant

The end-diastolic diameter (LVEDD) at day 30 post-DEC transplant was significantly higher in mice injected with fused MB^*wt*^/MB^*mdx*^ DEC cells (3.28 mm ± 0.31 mm, *p* = 0.009) and in mice injected with not-fused MB^*wt*^ + MSC^*mdx*^ (2.77 mm ± 0.16 mm, *p* = 0.034) cells when compared to the vehicle injected controls (2.33 mm ± 0.14 mm), although this effect was not observed at day 90 after DEC transplant (Table 1).

### Assessment of Functional Parameters of Ejection Fraction (EF) and Fractional Shortening (FS) Confirmed the Rebound Effect between Day 30 and Day 90 after Systemic DEC Transplant

**Fig. 5:**
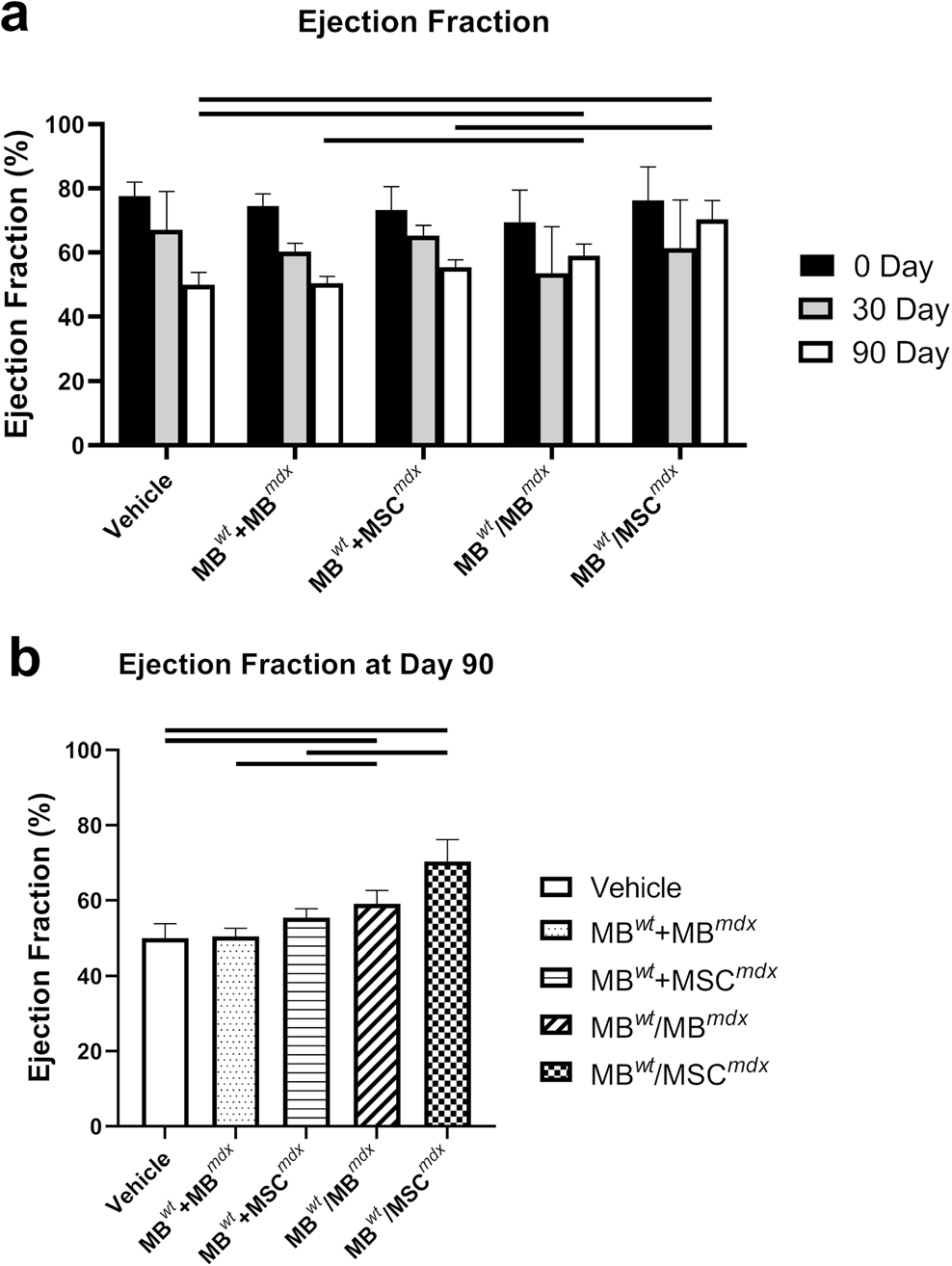
**Protective, rebound effect on the Ejection Fraction in*mdx*mice between 30 and 90 days after systemic-intraosseous DEC (MB**^***wt***^**/MB**^***mdx***^**DEC and MB**^***wt***^**/MSC**^***mdx***^**) transplant confirmed by Echocardiography**. **a** Ejection Fraction in *mdx* vehicle control and *mdx* mice systemically injected with not-fused (MB^*wt*^ + MSC^*mdx*^ and MB^*wt*^ + MB^*mdx*^) controls and MB^*wt*^/MSC^*mdx*^ or MB^*wt*^/MB^*mdx*^ DEC lines (*n* > 3). EF values revealed an increase between day 30 and day 90 for both DEC injected groups: MB^*wt*^/MB^*mdx*^ (day 30–53.51% ± 14.53%; day 90–59.07% ± 3.59% and MB^*wt*^/MSC^*mdx*^ (day 30–61.40% ± 15.01%; day 90–70.39% ± 5.80%), while a continued drop of EF was observed in the vehicle injected *mdx* controls (day 30–67.08% ± 12.00%; day 90–49.98% ± 3.82%). At day 90 MB^*wt*^/MB^*mdx*^ (*p* = 0.031) and MB^wt^/MSC^mdx^ (*p* = 0.049) groups demonstrated a higher ejection fraction compared to vehicle injected controls, and confirmed a rebound effect of the DEC therapy compared to day 30 values. b EF values for *mdx* mice injected with MB^*wt*^/MB^*mdx*^ (59.07% ± 3.59%) and MB^*wt*^/MSC^*mdx*^ (70.39% ± 5.80%) DEC cell lines were significantly increased at day 90 after DEC transplant compared to the not-fused control groups of MB^*wt*^ + MB^*mdx*^ (50.46% ± 2.08%, *p* = 0.007) and MB^*wt*^ + MSC^*mdx*^ (55.45% ± 2.29%, p = 0.049), respectively

**Fig. 6:**
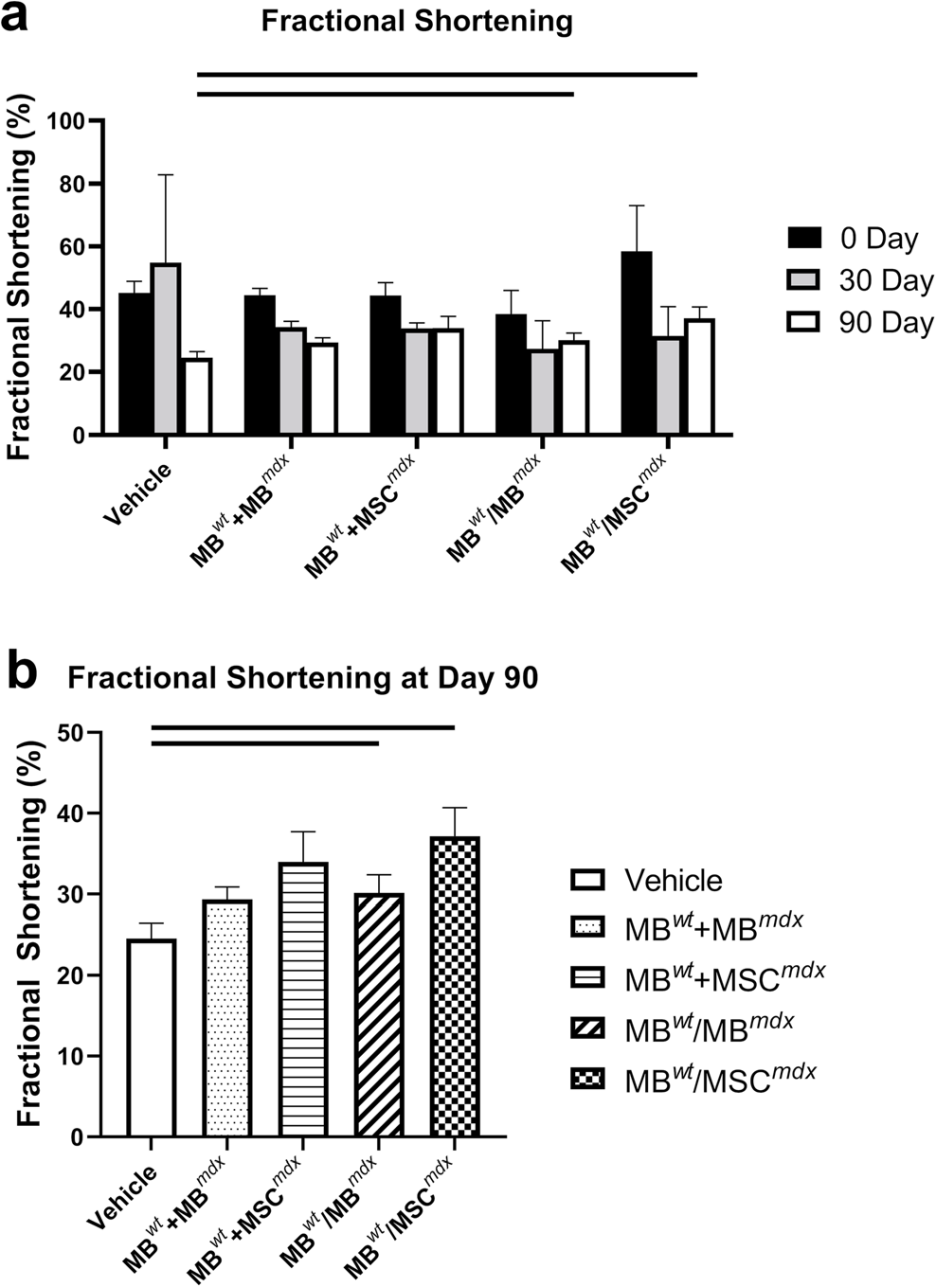
**Protective, rebound effect on the Fractional Shortening in*mdx*mice between 30 and 90 days after systemic-intraosseous DEC (MB**^***wt***^**/MB**^***mdx***^**DEC and MB**^***wt***^**/MSC**^***mdx***^**) transplant confirmed by Echocardiography. a** Fractional Shortening in *mdx* vehicle control and *mdx* mice systemically treated with not-fused (MB^*wt*^ + MB^*mdx*^ and MB^*wt*^ + MSC^*mdx*^) controls and MB^*wt*^/MB^*mdx*^ and MB^*wt*^/MSC^*mdx*^ DEC lines (n > 3). FS values revealed an increase between day 30 and day 90 after DEC transplant for both DEC lines, the MB^*wt*^/MB^*mdx*^ (day 30–27.35% ± 8.95; day 90–30.16% ± 2.22%) and MB^*wt*^/MSC^*mdx*^ (day 30–31.48% ± 9.35%; day 90–37.14% ± 3.56%), while a continued drop was observed in the vehicle injected *mdx* controls (day 30–54.90% ± 28.00%; day 90–24.55% ± 1.91%) **b** Fractional Shortening in *mdx* mice at day 90 after intraosseous DEC (MB^*wt*^/MB^*mdx*^ DEC and MB^*wt*^/MSC^*mdx*^) transplant showed significant increase in DEC injected mice when compared to vehicle injected *mdx* controls. FS values significantly increased for both, the MB^*wt*^/MB^*mdx*^ (30.16% ± 2.22%, *p* = 0.025) and MB^*wt*^/MSC^*mdx*^ (37.14% ± 3.56%, *p* = 0.045) DEC injected *mdx* mice compared to the vehicle injected controls (24.55% ± 1.91%)

At day 30 post-DEC transplant the assessed EF and FS values were not significant in any of the treatment groups compared to the vehicle injected control *mdx* mice. However, the values of EF and FS after an initial drop observed between day 0 to day 30 in the DEC treated groups, exhibited a rebound effect between day 30 and day 90 as evidenced by increase in the EF and FS values while the vehicle injected controls and groups injected with the not-fused cells showed further decline in the EF and FS across the same time points (Table 2, Figs. 5 and 6). In summary, the ejection fraction values revealed an increase between day 30 and day 90 for both DEC injected groups of: MB^*wt*^/MB^*mdx*^ (day 30–53.51% ± 14.53%; day 90–59.07% ± 3.59% and MB^*wt*^/MSC^*mdx*^ (day 30–61.40% ± 15.01; day 90–70.39% ± 5.80%), while a continued drop of EF was observed in the vehicle injected *mdx* controls (day 30–67.08% ± 12.00%; day 90–49.98% ± 3.82%).

Fractional shortening values revealed an increase between day 30 and day 90 after DEC transplant for both, the MB^*wt*^/MB^*mdx*^ (day 30–27.35% ± 8.95; day 90–30.16% ± 2.22%) and MB^*wt*^/MSC^*mdx*^ (day 30–31.48% ± 9.35%; day 90–37.14% ± 3.56%) DEC lines, while a continued drop was observed in the vehicle injected *mdx* controls (day 30–54.90% ± 28.00%; day 90–24.55% ± 1.91%) (Fig. 6a).

### Ejection Fraction (EF) and Fractional Shortening (FS) Significantly Increased in DEC Treated *mdx* Mice Compared to the Vehicle Injected Controls at 90 Days after Systemic DEC Transplant

The echocardiography values assessed at day 90 for the ejection fraction (EF) for the MB^*wt*^/MB^*mdx*^ (59.07% ± 3.59%, *p* = 0.031) and MB^*wt*^/MSC^*mdx*^ (70.39% ± 5.80%, *p* = 0.049) injected DEC lines were found to be significantly increased compared to the vehicle injected *mdx* controls (49.98% ± 3.82%) (Fig. 5b). In addition, assessment of the fractional shortening (FS) was significantly increased for both the MB^*wt*^/MB^*mdx*^ (30.16% ± 2.22%, *p* = 0.025) and MB^*wt*^/MSC^*mdx*^ (37.14% ± 3.56%, *p* = 0.045) injected *mdx* mice compared to the vehicle injected controls (24.55% ± 1.91%)(Fig. 6b). In addition, the ejection fraction values for *mdx* mice injected with MB^*wt*^/MB^*mdx*^ and MB^*wt*^/MSC^*mdx*^ DEC cell lines were also significantly increased compared to the not-fused control groups of MB^*wt*^ + MB^*mdx*^ (50.46% ± 2.08%, *p* = 0.007) and MB^*wt*^ + MSC^*mdx*^ (55.45% ± 2.29%, p = 0.049), respectively at 90 days after DEC transplant. The other parameters were not found to be significant at day 90 after DEC transplant.

## Discussion

Cardiomyopathy is the most devastating cause of morbidity and mortality in Duchenne Muscular Dystrophy (DMD) patients, and affects 30% of patients by 14 years of age, 50% of patients by 18 years, and is diagnosed in all older surviving patients [51]. Heart failure in these DMD patients is the result of cardiac myocyte death and fibrosis, leading to both diastolic and systolic dysfunction. This manifests clinically as the stiff ventricles that cannot exhibit compliance in response to the blood delivered from the atria due to the disarrayed muscle fibers. The inability to eject blood from the ventricles into the systemic circulation, results in a reduced ejection fraction [4, 8, 9, 51]. In the DMD mouse model, cardiac hypertrophy has been reported to develop as early as 16–21 weeks of age, correlating to an increase in cardiomyocyte size seen on hematoxylin and eosin staining and to ventricular wall fibrosis seen on Masson’s trichrome staining [23].

Recent echocardiographic analysis of the *mdx* mouse model by Bondoc et al. have revealed a significant drop in ejection fraction as the *mdx* mouse ages from 6 to 8 weeks to 15–17 weeks old in comparison to the wild type [10]. Furthermore, an increase in stroke volume is seen in a healthy wild type mouse between the same time points, while *mdx* mice do not show any difference in stroke volume as the mouse ages [10, 23]. Similar to Bondoc et al. and Fayssoil et al., we have not observed changes in stroke volume in the *mdx* vehicle mice as they aged over the 90 days. As in the Bondoc et al. report, our findings confirmed a continued drop in ejection fraction in the *mdx* vehicle injected mice across the same time points as assessed in our study (day 0 to day 90) [9, 10]. However, mice treated with MB^*wt*^/MB^*mdx*^ or MB^*wt*^/MSC^*mdx*^ DEC lines cells exhibited a protective effect that was significant at day 90 post-transplant with the ejection fraction values being 10% and 20% higher, respectively, compared to vehicle injected controls.

During systole, the left ventricle contracts radially, circumferentially, and longitudinally. Fractional shortening is a measure of cardiac contractility expressed as the percentage by which the left ventricle reduces in size longitudinally and is defined by the fractional shortening formula above. Longitudinal function has been shown to be an early marker of left ventricular diastolic dysfunction, and typically precedes a drop in ejection fraction [3–5, 8]. Geometric changes such as increased relative ventricular wall thickness may allow for preserved ejection fraction in the setting of diastolic heart dysfunction despite depressed myocardial shortening in two orthogonal planes [4, 8].

In *mdx* mice, it is known that posterior wall thickness increases secondary to myocardial fibrosis as the pathology progresses, which may reflect an adaptation to early diastolic dysfunction. However, once the ventricular wall stiffens reaches the point where it can no longer be compliant with the blood from the atria, the diastolic dysfunction starts to worsen. This leads to systolic dysfunction and large drops in ejection fraction as heart failure symptoms become more prominent [2, 3, 9].

In our study, fractional shortening demonstrated a large initial drop between day 0 to day 30 across all groups. This parameter continued to drop in the *mdx* vehicle injected mice until day 90 and the values were comparable with the Fayssoil et al. 2013 studies examining the long-term function of the untreated *mdx* mice using echocardiography [9]. However, in our study FS in mice treated with both MB^*wt*^/MB^*mdx*^ and MB^*wt*^/MSC^*mdx*^ DEC cells exhibited a protective effect that was significantly improved compared to the vehicle injected *mdx* controls at day 90. FS demonstrated a rebound effect between day 30 and day 90 in MB^*wt*^/MB^*mdx*^ and MB^*wt*^/MSC^*mdx*^ DEC cell treated mice, while this effect was not observed in mice injected with vehicle and with not-fused MB^*wt*^ + MB^*mdx*^ and MB^*wt*^ + MSC^*mdx*^ cells, suggesting that the fused cells are more efficacious in counteracting the effects of systolic dysfunction in *mdx* mice due to better engraftment potential and lower levels of rejection. In our study, mice treated with not-fused cells also had lower values for morphometric parameters for LVEDV and LVESV than DEC treated groups at day 30, and then had lower EF and FS values than DEC treated groups at day 90, while morphometric parameters stabilized to levels observed in the vehicle injected *mdx* controls. This could be indicative of early diastolic dysfunction in mice treated with not-fused cell as evidenced by a stiffer less compliant heart undergoing greater myocardial fibrosis, which then translated to the systolic dysfunction as evidenced by a decreased EF and FS as the mice aged over our 90 day period of observation.

Large initial drops in both, the ejection fraction and fractional shortening are seen across all treatment groups from day 0 to day 30, suggesting that there is a therapeutic latency in response to the treatments and showing evidence of a protective effect on the cardiac parameters and restoration of cardiomyocyte dystrophin to clinically appreciable levels. Based on the ability of myoblasts to fuse, DEC cells after injection could spontaneously fuse with the recipient myoblasts, and thus establish and maintain dystrophin expression at higher levels. In the skeletal muscle, dystrophin levels should be maintained between 10 and 20% of wild type levels to trigger sustained skeletal muscle force improvement in the *mdx* mice [37]. Mesenchymal stem cells have been shown to restore dystrophin levels up to 4% in skeletal myocytes of irradiated *mdx* mice at 12 weeks, with 10–30% of *mdx* cells containing fused donor nuclei confirmed by DAPI staining [24]. Our previous studies have demonstrated that DEC cells created via ex-vivo fusion of wild type myoblasts and *mdx* mouse myoblasts restore dystrophin levels up to 37.27% after DEC transplant to the dystrophin-deficient *mdx* mice [32]. Subsequent in vivo gastrocnemius muscle testing in *mdx* mice after DEC injection showed improvement in muscle strength and function which correlated with increased levels of dystrophin expression [32].

Other notable trends in our data include an increase in heart rate as the *mdx* mouse ages, regardless of the treatment group. There were also larger end-diastolic and end-systolic volumes found in the *mdx* mice within the same treatment group as they aged. This is consistent with study by Spurney et al. involving *mdx* mouse echocardiography, which affirmed that as the mice age, the heart will have to move a higher volume of blood around the body [5]. Although stroke volume did not increase in response to DEC treatments, the rebound effect of fractional shortening does suggest an improvement in cardiac contractility and is responsible for the improvement seen in ejection fraction.

This is the first study assessing the cardiac effects of DEC therapy at 90 days after systemic injection to the *mdx* mice. Assessment of changes occurring in cardiac muscle in *mdx* mouse model suggested that restoration of cardiac myocyte dystrophin levels as low as 4–15% of the wild type mice can delay or even partially ameliorate the effects of cardiomyopathy in the *mdx* mice [24]. Immunofluorescence assessment of the heart muscle samples at 90 days after systemic DEC transplant confirmed restoration of dystrophin expression in the MB^*wt*^/MB^*mdx*^ (15.73 ± 5.70, *p* < 0.0001) and MB^*wt*^*/*MSC^*mdx*^ (5.22% ± 1.10, *p* = 0.0406) DEC injected mice when compared to the vehicle injected *mdx* controls (2.01% ± 1.36). This correlated with the cardiac protection and rebound effect seen on echocardiography analysis at day 90 in these respective DEC treatment groups**.**

Determining the ideal routes of administration for various DMD therapies have proven to be a challenge [11, 12]. Though there is scarce literature comparing routes of stem cell administration in the *mdx* mice for treatment of DMD cardiomyopathy, studies using a murine model for Ashermann’s syndrome have shown systemic administration of bone-marrow derived stem cells led to better stem cell engraftment in uterus, leading to improved fertility when compared with local intrauterine injection [53]. Based on our experience, with the systemic-intraosseous injection of bone marrow cells and chimeric cells showing better engraftment and maintenance of the donor derived chimerism [43, 45, 46], in this study, we focused on systemic administration of DEC through intraosseous injection, as this facilitates the process of systemic delivery and potential engraftment to other muscles including the cardiac muscle as confirmed in this study by increased dystrophin expression and reduced fibrosis which correlated with significant improvement of EF and FS on echocardiography. In contrast, in our previous study, following intramuscular administration of DEC only local effect was seen in the injected muscles [31, 32]. In addition to affecting the cardiac muscle, DMD leads to global weakness in all skeletal muscles, including muscles important for essential functions such as respiration - intercostal muscles and diaphragm - and muscles involved in swallowing and mastication [54]. Therefore, a systemic route of DEC delivery would be clinically preferred method, since local intramuscular injections are a lengthily procedures performed in pediatric population under general anesthesia and are addressing only few selected muscles without addressing the systemic problems such as e.g. cardiomyopathy affecting children with DMD.

Corticosteroids have been shown to prolong cardiopulmonary survivorship in DMD human patients, the exact mechanism by which this occurs is unknown, and long term use of corticosteroids have unintended negative side effects. In addition, in the mdx model, steroids have even shown to expedite the decline of cardiac function secondary to increased end diastolic pressure, increased myocardial fibrosis, and decreased stroke volume [13]. Further studies examining DEC therapy as a stand-alone or as an adjunct to pharmaceuticals that act on the angiotensin-renin system may be warranted [11, 13]. Initiating antifibrotic therapies before the development of overt LV systolic dysfunction may be helpful in delaying DMD related cardiac dysfunction, so coupling DEC therapy treatments with angiotensin-converting enzyme inhibitors, aldosterone antagonists, or angiotensin receptor blockers should also be investigated in *mdx* mice also be called for [11, 13, 14]. Analysis with imaging designed to quantify myocardial fibrosis with echocardiographic measurements or with late gadolinium enhancement studies in addition to histopathology is also warranted within such studies [9, 10, 52, 55].

There are several limitations of the study that should be addressed. We have based our study on other researchers reports on the echocardiography [56] or cardiac MRI [57] where numbers of animals (*n* = 3 or *n* = 4) were similar to our study.

Echocardiographic measurements in small animal models such as mice are challenging due to the unexpected technical issues, anatomical issues or potential animal loss due to the repeated administration of anesthesia during multiple echo assessments at different time-points of the study. Echocardiography measurements of LV function assume a uniform, global LV function without regional wall motion abnormalities, a symmetric LV shape, and constant loading conditions for preload and afterload**.** However, in small animal models there are other factors which may affect these measurements and these include the fact that mice can have different heart shapes, heart sizes, and different function at a given longitudinal heart strains due differences in cardiac fibrosis patterns as the disease progresses [58]. Reproducibility of echocardiographic measurements and operator dependence, with regard to intra-observer validity should be also considered specifically in small animals. We have addressed the issue of reproducibility of measurements and dedicated one trained in echocardiography researcher to perform echocardiography and to collect all echo data at day 0, day 30, and day 90. The technician performing the echo tracings on VEVO, who was different than the researcher who performed actual on echos, was blinded from the treatment groups, which helped reduce some bias. The echocardiography assessments and the obtained results were found to be within the expected ranges and comparable with other studies on cardiomyopathy assessed in the mdx mice [59]. Finally, administration of anesthesia affects ability to control preload, as the heart rate and blood pressure fluctuate dramatically with small adjustments made during the administration of anesthesia. We attempted to maintain heart rate at a target of 350 and 400 beats per minute, but there were times that the mice would still not be adequately sedated, which required us to give more anesthesia, which could lead to variations in preload and afterload. However, as reported by Bostick et al. the values of pressure-volume (P/V) loops for 22-month *mdx* male mice were stable, showing no obvious shift, suggesting no differences in preload between the chosen model animals [60]. In addition, as previously reported by Roth et al. [61] and confirmed by Fayssoil et al. [9] anesthetic agents may have detrimental effect on heart function in mouse models. Isoflurane, applied in our study, decreases contractility and heart rate in mice but carries lower risk of cardiac depression in comparison to other drugs (ketamine, xylazine) and in prolonged studies is clearly preferable.

Carrying future studies to longer time points after administration of treatments will be vital in assessing the sustainability of these responses and the long-term viability of novel DEC cell therapies as a potential clinical treatment options for in Duchenne’s patients.

In summary, to the best of our knowledge this is the first report assessing cardiac function at 90 days after systemic DEC transplant to the *mdx* mice. We have confirmed the protective effect of DEC on cardiac function shown by increased values of the ejection fraction and fractional shortening, which at 90 days revealed the rebound effect when compared to the vehicle injected controls and mice receiving not-fused cell therapy. Moreover, these functional improvements correlated with restoration of dystrophin expression in cardiac muscle at 90 days post-DEC transplant. This study establishes DEC as a promising new option for cardiac protection and potential amelioration of the DMD related cardiac pathology.
